# Real-time free-breathing strategy for tracking labeled cells with in-vivo vardiac MRI

**DOI:** 10.1186/1532-429X-11-S1-P31

**Published:** 2009-01-28

**Authors:** Yijen L Wu, Cornelius Brinegar, Zhi-Pei Liang, Qing Ye, T Kevin Hitchens, Lesley M Foley, Chien Ho

**Affiliations:** 1 Pittsburgh NMR Center for Biomedical Research, Carnegie Mellon University, Pittsburgh, PA USA; 2 Electric and Computer Engineering, University of Illinois at Urbana-Champaign, Urbana-Champaign, IL USA

**Keywords:** Cine Imaging, Short Acquisition Time, Heterotopic Transplantation, Temporal Resolution Data, High Temporal Resolution Data

## Introduction

Non-invasive *in vivo* cellular imaging with MRI has greatly broadened our understanding in many cellular and molecular processes, as well as in monitoring stem cell therapy. The target cells can be made MRI-detectable by labeling them with iron-oxide contrast agents, such as micrometer-sized-iron-oxide (MPIO) particles. With sufficient iron loading per cell, it is possible to detect single cells *in vivo* with MRI. However, motion artifacts and the need for gated acquisition have made imaging cells in the heart more challenging and time-consuming. The goal of this study is to develop a strategy to enable real-time imaging of individual labeled cells without the need for a gated or breath-hold protocol. To this end, we have implemented a new model-based imaging scheme for high-resolution free-breathing imaging, using the Partially Separable Function (PSF) model.

## Methods

### 1. Model system

We use a rodent abdominal heterotopic working heart and lung transplantation model with DA to BN rat pair, using BN-to-BN transplantation as isograft controls.

### 2. Iron oxide particle labeling

Immune cells, mostly macrophages, were labeled *in vivo* by direct intravenous injection of 3 mg micrometer-sized-iron-oxide (MPIO) particles 1 day prior to MRI scans.

### 3. MRI protocols

Real-time free-breathing imaging, and ECG and respiration gated T_2_* and cine imaging were performed using a Bruker AVANCE 4.7-T system with a home-built 5.5 cm surface coil and an in-plane resolution of 195 μm.

### 4. Partially Separable Function (PSF) method and theory

The PSF model approximates the hypersurface, *d*(*k*, *t*), formed by the time varying data as a summation of *M* simpler functions that are each the product of one-dimensional functions *α*_*m*_(*k*) and *ϕ*_*m*_(*t*), and recent results have shown that cardiac imaging with breathing exists in a low rank space where *M* = 16. Data collection consists of interleaving two scans: (i) a high temporal resolution data set for estimating {*ϕ*_*m*_(*t*)}^*M*^_*m* = 1_and (ii) a high spatial resolution scan used to estimate {*α*_*m*_(*k*)}^*M*^_*m* = 1_. The low rank of the model allows for temporal interpolation over time intervals failing the Nyquist criterion thereby avoiding the need to time data collection with the cardiac and respiratory motions.

## Results

We have used a rodent heterotopic transplantation model for tracking labeled immune cells *in vivo*. In this model, immune cells, mostly macrophages, can be labeled *in vivo* by direct intravenous administration of MPIO particles; labeled immune cells then migrate to the rejecting transplanted hearts and are detected by T_2_*-weighted imaging. Low-dosage MPIO particles are used in this study for sparse labeling of macrophages to promote the visualization of single cells, even though the images lack the necessary spatial resolution by at least a factor of 4.

**Figure 1:**
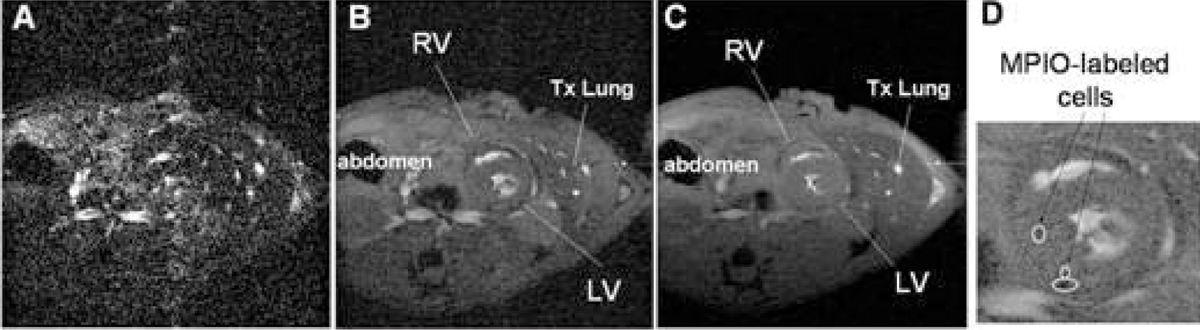
**(A) Fourier reconstruction of free-breathing MRI**. (B) Model-based reconstruction of the same free-breathing MRI. (C) ECG-and-respiration-gated cine imaging for comparison. (D) Enlarged view of the heart showing the infiltrated MPIO-labeled cells.

Without gating, real-time free-breathing MRI results in blurring due to motion artifacts (Fig. 1A). The model-based data collection and reconstruction scheme (Fig. 1B) yields a high-quality image comparable to ECG-and-respiration-gated cine imaging (Fig 1C), however in a much shorter acquisition time. The model-based method enables identification of MPIO-labeled cells *in vivo* (Fig. 1D) in real-time data acquisition. The model-based method acquires data continuously whereas the gated scheme collects data intermittently, depending upon the relationship of the ECG and respiratory signals. In addition to a shorter acquisition time, the continuous model-based scheme preserves all information in the *k-t* space, which is especially important in functional evaluation of the diseased conditions with arrhythmia or compromised breath-holding.

## Conclusion

The model-based imaging scheme allows real-time free-breathing imaging with resolution and image quality sufficient for tracking labeled cells *in vivo* in beating hearts.
